# Supplementary material for: Low-temperature anode-free potassium metal batteries
Source: Nat Commun. 2023 Sep 26;14:6006. doi: 10.1038/s41467-023-41778-6 (PMC10522645; doi:10.1038/s41467-023-41778-6)
Supplement: Supplementary file 1 — Supplementary Information File [file 41467_2023_41778_MOESM1_ESM.pdf]

Supplementary information

Low-Temperature Anode-Free Potassium Metal Batteries

Mengyao Tang[1,3], Shuai Dong[1,3], Jiawei Wang[1], Liwei Cheng[1], Qiaonan Zhu[1], Yanmei Li[2], Xiuyi Yang[1], Lin Guo[1*] and Hua Wang[1*]

[1]School of Chemistry, Beijing Advanced Innovation Center for Biomedical Engineering, Beihang University, Beijing, China

[2]School of Materials Science and Engineering, University of Science and Technology Beijing, Beijing, China

[3]These authors contributed equally: M. Tang, S. Dong.

***Correspondence: wanghua8651@buaa.edu.cn; guolin@buaa.edu.cn**

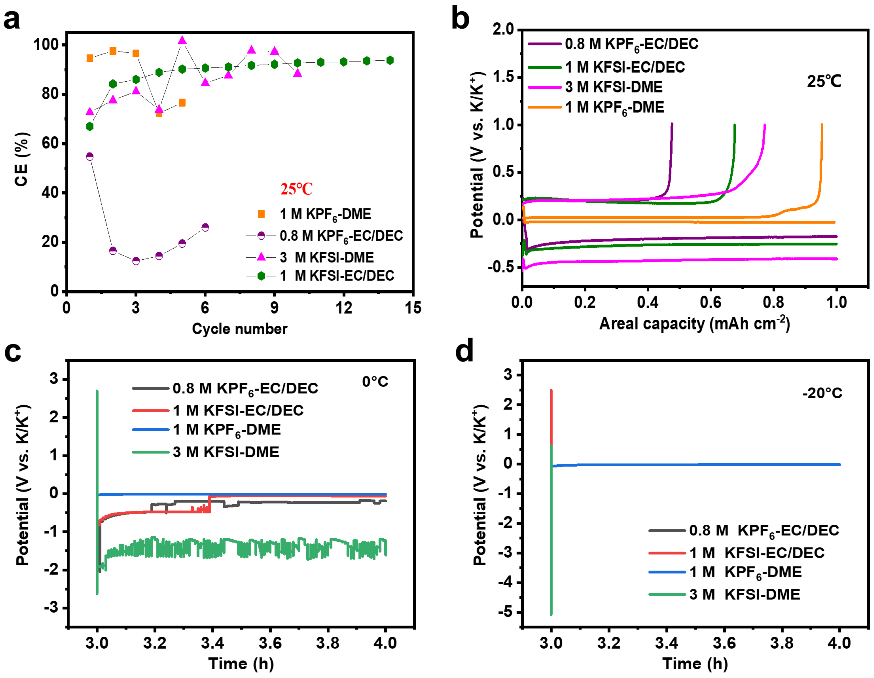

Supplementary Figure 1. (a) CE tests of the K||Cu half cells cycled with different electrolytes at 1.0 mA cm^{-2}/1 mAh cm^{-2} (25°C); (b) The corresponding voltage profiles of K plating/stripping in the first cycle; Voltage profiles of K plating on Cu current collectors at (c) 0°C and (d) -20°C after resting for 3 h.

It can be found that the K||Cu half cells with 1 M KPF_6-DME exhibit the highest initial CE and lowest electrochemical polarization, but a poor cycle life of 3 cycles at 25°C. With the temperature decreased to 0 and -20°C, the K||Cu half cells only with 1 M KPF_6-DME electrolyte can work with a small overpotential during the plating process.

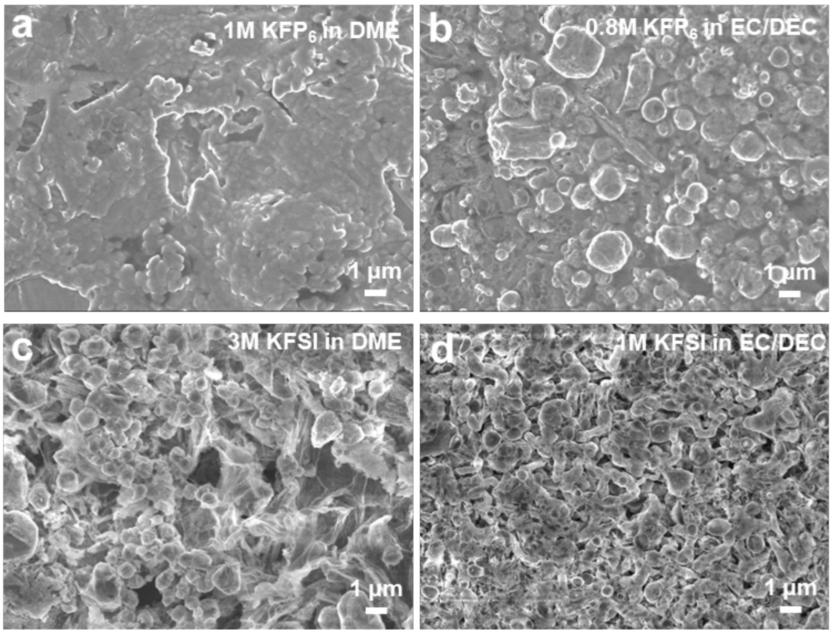

Supplementary Figure 2. SEM images of the K deposited layer on the Cu current collector with 4 electrolytes at 1 mA cm^{-2}/1 mAh cm^{-2} and 25°C.

For 1 M KPF_6-DME, a relatively tense and uniform K deposit layer on the Cu substrate is observed, favoring the high initial CE of K||Cu cells.

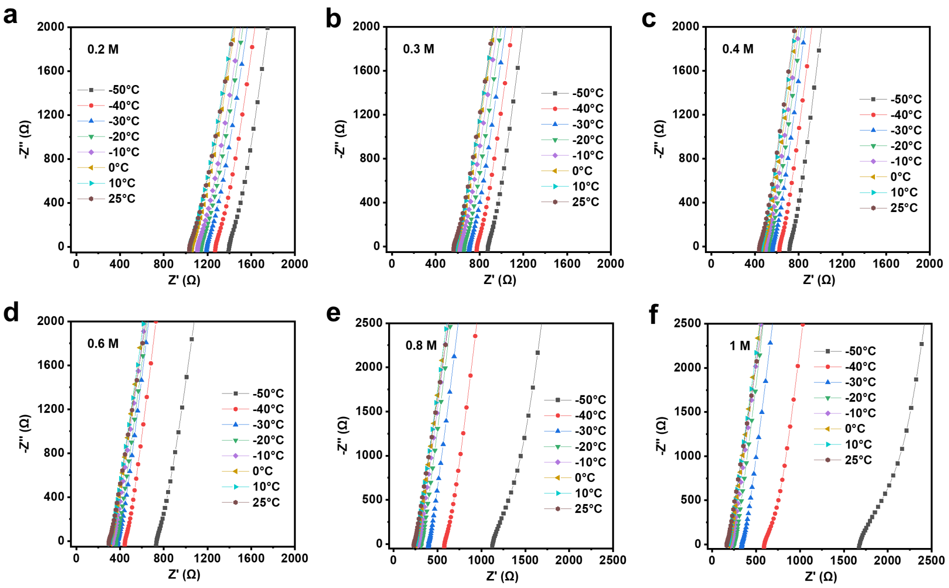

Supplementary Figure 3. (a-f) Nyquist plots of the KPF_6/DME electrolytes with various concentrations at different temperatures.

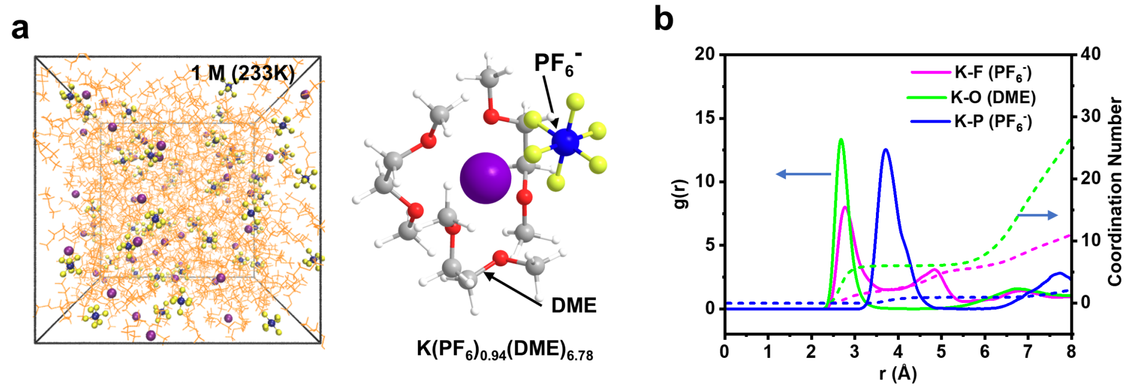

Supplementary Figure 4. (a) The Snapshots of the MD simulation boxes of 1 M KPF_6-DME at 233K; (b) The corresponding RDF data.

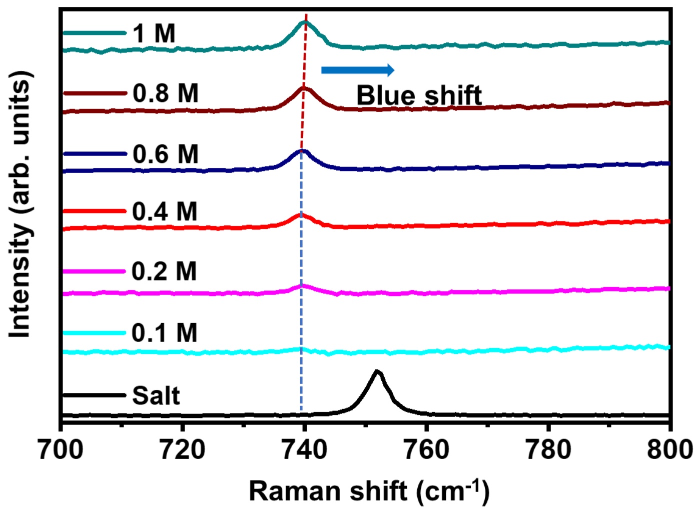

Supplementary Figure 5. Raman spectra of KPF_6/DME electrolytes with different concentrations at -40℃.

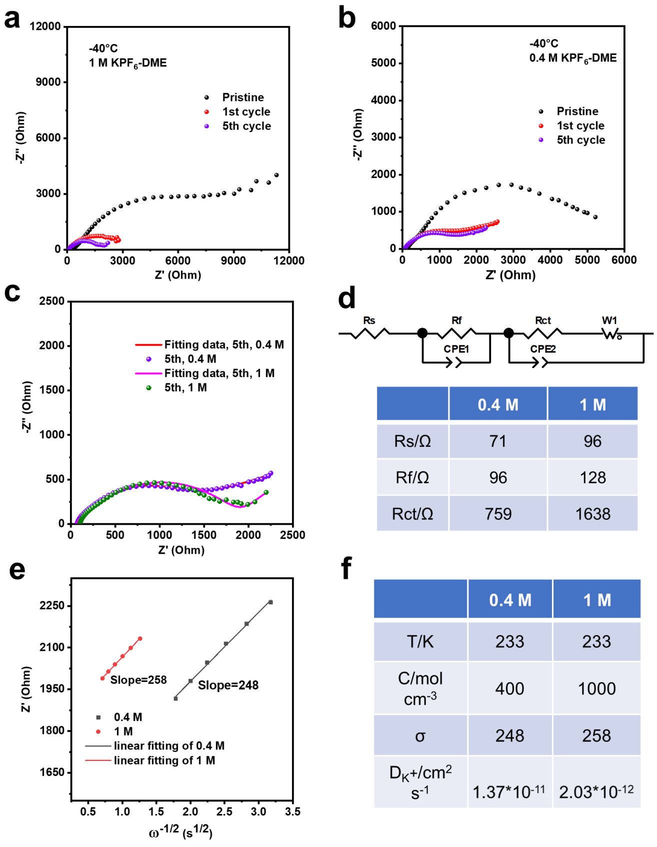

Supplementary Figure 6. EIS tests of K||Cu cells in (a) 0.4 M and (b) 1 M KPF_6-DME, respectively, at -40°C; (c) Fitted Nyquist plots at 5th cycle; (d) Equivalent circuit model and the corresponding impedance data; (e) The plot of Z'-$\omega^{-1/2}$; (f) The calculated K^+ diffusivity. The corresponding impedance data is shown in **Supplementary Table 3**.

After 1st cycle activation process, the impedance of K||Cu cells exhibits an obvious decrease both in 0.4 and 1 M KPF_6-DME at -40°C. Considering the interface reaction limited by the low temperature, we then increase cycle number to 5 to form a SEI with relatively stable composition. Based on the fitted results of 5th cycle, 0.4 M KPF_6-DME exhibits a low charge-transfer resistance (Rct) of 759 Ω compared with 1 M KPF_6-DME (Rct=1638 Ω), demonstrating the decreased energy barrier for K^+ ion-desolvation process.

The K^+ diffusivity is calculated by the equation[1,2]:

$$D_{K^+} = \frac{2R^2T^2}{n^4F^4\sigma^2A^2C^2}$$

, where *R, T, A, F, n, σ* and *C* are the gas constant of 8.314 J K^{-1} mol^{-1}, the absolute temperature of 233 K, the surface area of the electrode of 0.8 cm^{-2}, the Faraday's constant of 96500 C mol^{-1} , the number of electrons per molecule, Warburg coefficient and the K^+ concentration, respectively. Warburg coefficient is the slope of Z'-$\omega^{-1/2}$.

From the above analysis, the calculated D_{K^+} is 1.37*10^{-11}, 2.03*10^{-12} cm^2 s^{-1} for 0.4 and 1 M KPF_6-DME, respectively, at -40°C.

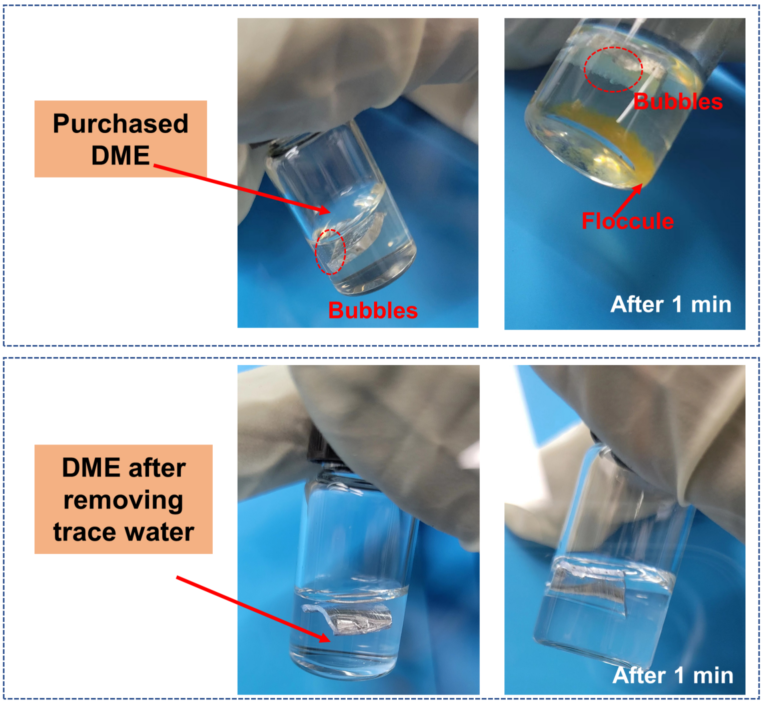

Supplementary Figure 7. Digital photographs of reaction phenomenon between K and trace water in DME.

When K metal was soaked in purchased DME (Sigma-Aldrich, anhydrous, 99.5%, inhibitor-free), an obvious chemical reaction between K and H_2O occurred, accompanied by large number of bubbles and yellow flocculent precipitates. After the reaction, the completely anhydrous DME was obtained by collecting the supernatant liquid. Then, we repeated the above-mentioned tests, and found that DME became more stable with K metal, which means that K mainly reacts with water rather than DME. Thus, it is a logical treatment to remove the residual water in DME by K metal.

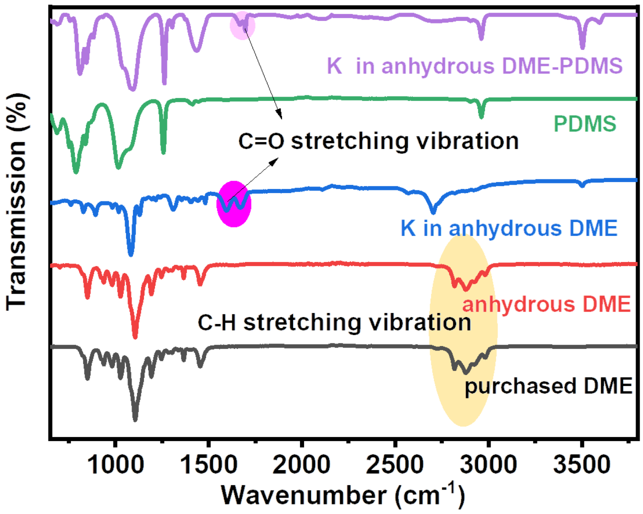

Supplementary Figure 8. ATR-FTIR spectra of decomposition products on K metal surface.

After removal of water by K metal, the obtained anhydrous DME can still react with K. From the ATR-FTIR spectra, the C=O stretching vibration peaks at 1605 and 1679 cm^{-1} were clearly detected on the K surface, which can be attributed to the decomposition product (CH_3COO^-) from DME molecule[3]. When PDMS added in the anhydrous DME, the intensity of C=O obviously decreased, demonstrating that PDMS can suppress the decomposition of DME on K metal surface.

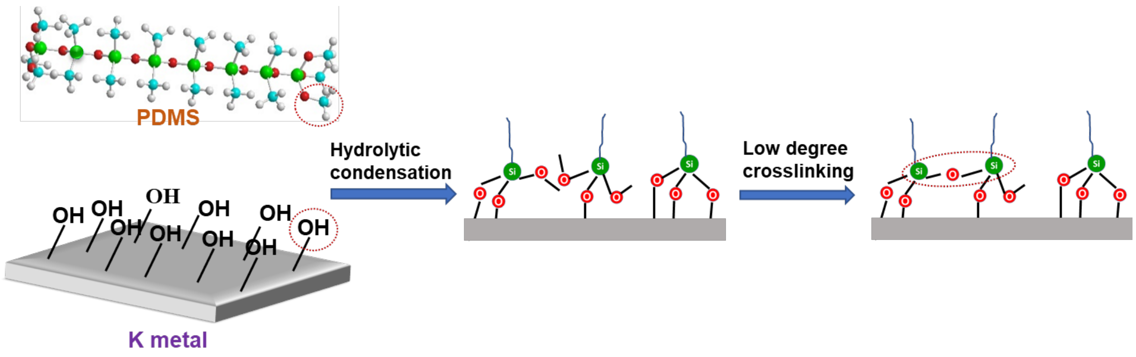

Supplementary Figure 9. Schematic representation of the interaction mechanism between K and PDMS.

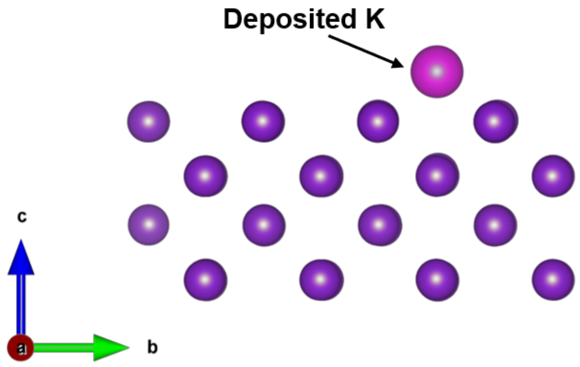

Supplementary Figure 10. The structure model of K atom deposited on K (001) substrate.

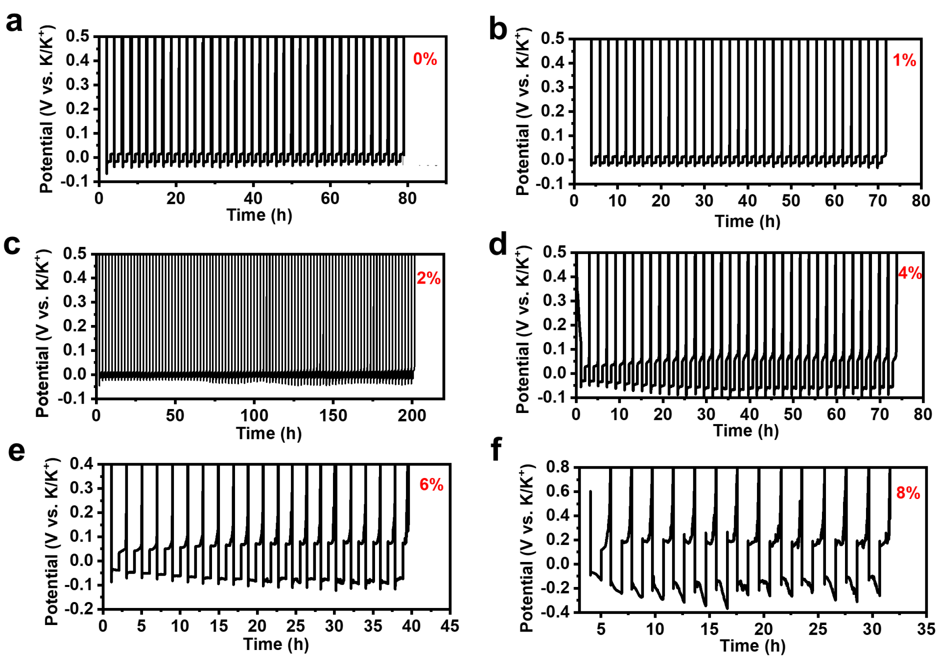

Supplementary Figure 11. K plating/stripping tests by K||Cu half cells in 0.4 M KPF_6-DME with various PDMS contents at 1.0 mA cm^{-2}/1 mAh cm^{-2} and -40°C.

When the PDMS content increased to 4, 6 and 8 vol. %, it can be found that the electrochemical polarization of Cu||K batteries became more serious and its cycling stability was even worse, owing to the PDMS with a high molecular weight of 770 can increase viscosity and decrease ionic conductivity of electrolytes.

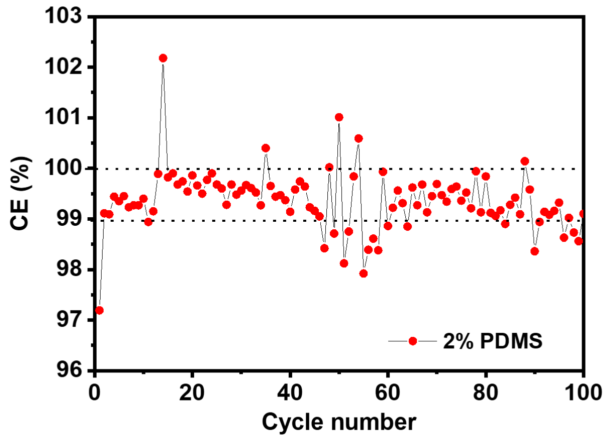

Supplementary Figure 12. The enlarged plot of CE values with 2 vol. % PDMS at -40°C.

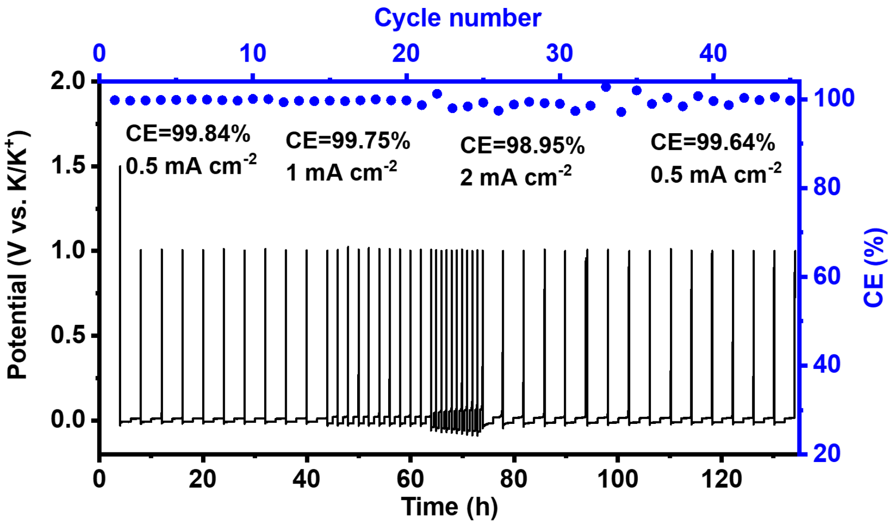

Supplementary Figure 13. The rate capability test of K||Cu cells at 1 mAh cm^{-2} and -40°C.

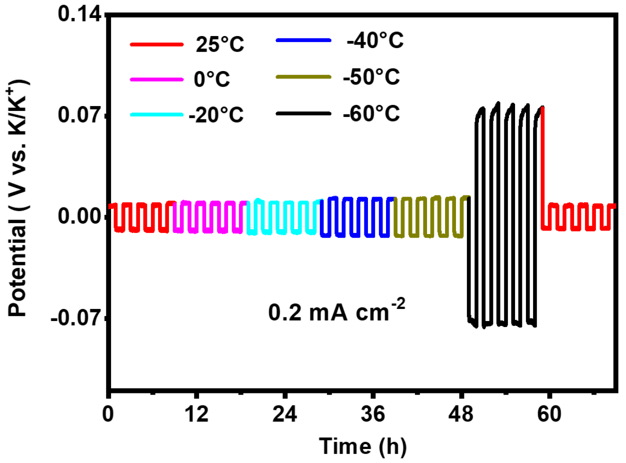

Supplementary Figure 14. The temperature-dependent electrochemical performance of K||K cells with the KDP electrolyte at 0.2 mA cm^{-2}/0.2 mAh cm^{-2}.

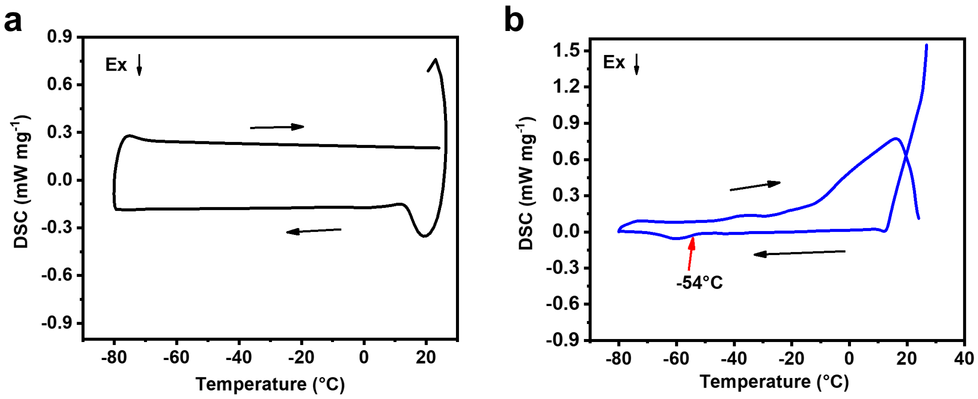

Supplementary Figure 15. The DSC tests of (a) PDMS and (b) the KDP electrolyte.

The freezing point of PDMS (trimethylsiloxy terminated, M.W. 770, Alfa) is < -40°C from Material Safety Data Sheet (MSDS). Besides, its phase transition is not detected until −80°C based on the DSC test. Thus, PDMS should be a good cryogenic additive.

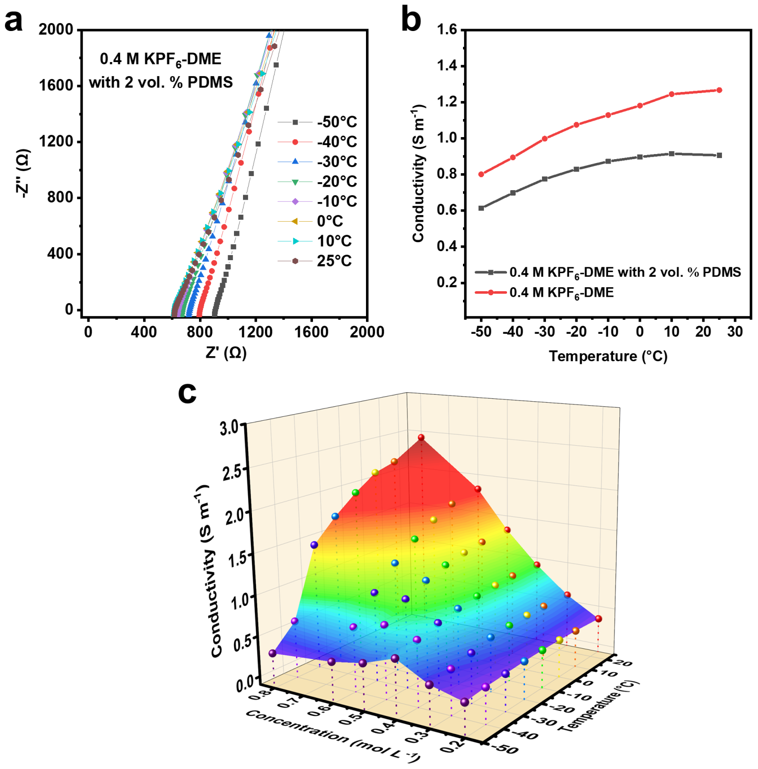

Supplementary Figure 16. (a) The Nyquist plots of the 0.4 M KPF_6-DME with 2 vol. % PDMS (KDP) at different temperatures; (b) Temperature-dependent ionic conductivity of 0.4 M KPF_6-DME electrolyte with and without PDMS additive; (c) Temperature-dependent ionic conductivity of the KDP electrolyte with a series of concentration gradients.

The introduction of PDMS into the 0.4 M KPF_6-DME electrolyte leaded to a slight decrease in ionic conductivity from 0.89, 0.80 S/m to 0.69, 0.61 S/m at -40 and -50°C, respectively. Moreover, 0.4 M electrolyte still achieved the highest ionic conductivity at -50°C among a series of concentration gradients.

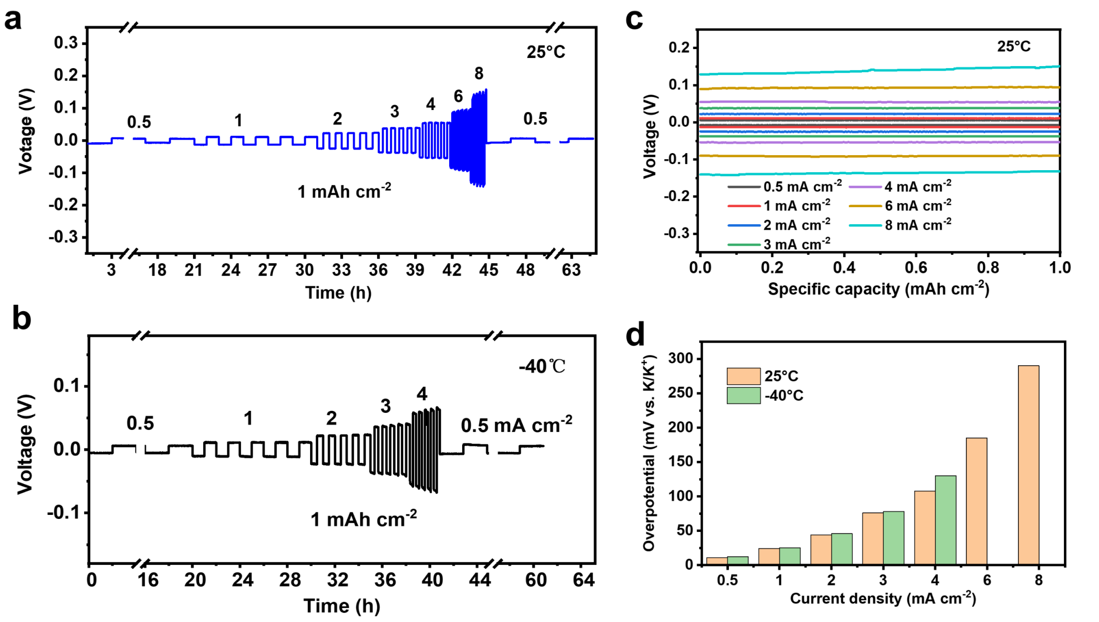

Supplementary Figure 17. The rate performance of K||K cells with the KDP electrolyte at (a) 25°C and (b) -40°C, respectively; (c) Representative plating/stripping curves at different current densities and 25°C; (d) Comparison of overpotentials obtained from (a) and (b).

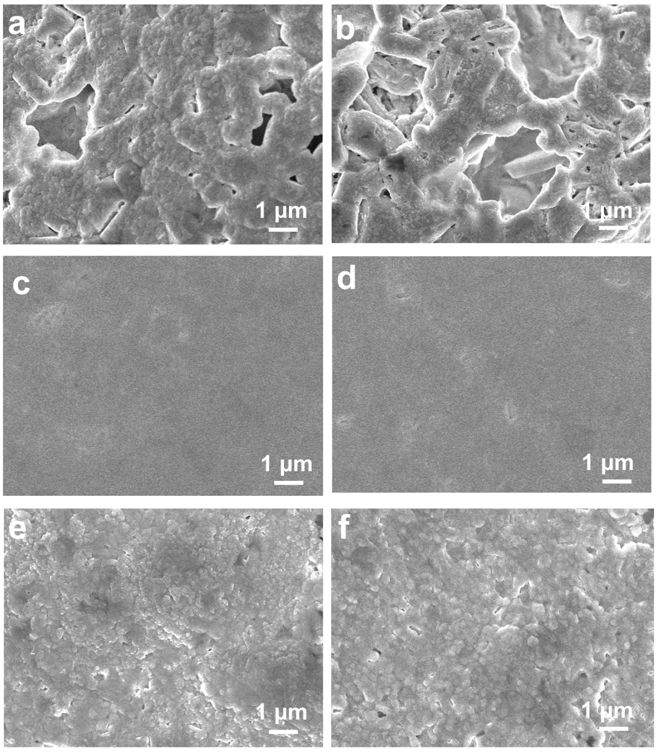

Supplementary Figure 18. SEM images of K metal anode after 10 cycles at -40°C: 0.4 M KPF_6-DME electrolyte at (a) 1 mA cm^{-2}/1 mAh cm^{-2} and (b) 2 mA cm^{-2}/1 mAh cm^{-2}; the KDP electrolyte at (c) 1 mA cm^{-2}/1 mAh cm^{-2} and (d) 2 mA cm^{-2}/1 mAh cm^{-2}. SEM images of K metal anode with the KDP electrolyte after 100 cycles at (e) 1 mA cm^{-2}/1 mAh cm^{-2} and (f) 2 mA cm^{-2}/1 mAh cm^{-2}, respectively (-40°C).

After 10 cycles at -40°C, the K||K cells were then disassembled to observe the morphology of the K anodes. When 0.4 M KPF_6-DME electrolyte was used, extremely porous K was observed (Supplementary Figure 18a,b). In contrast, the PDMS system yielded smooth K metal surface (Supplementary Figure 18c,d). Even after 100 cycles, the relatively uniform K surface morphologies were still observed, confirming that the PDMS can be conducive to improve the interfacial stability and serve as a potassiophilic interface to guide uniform K deposition (Supplementary Figure 18e,f).

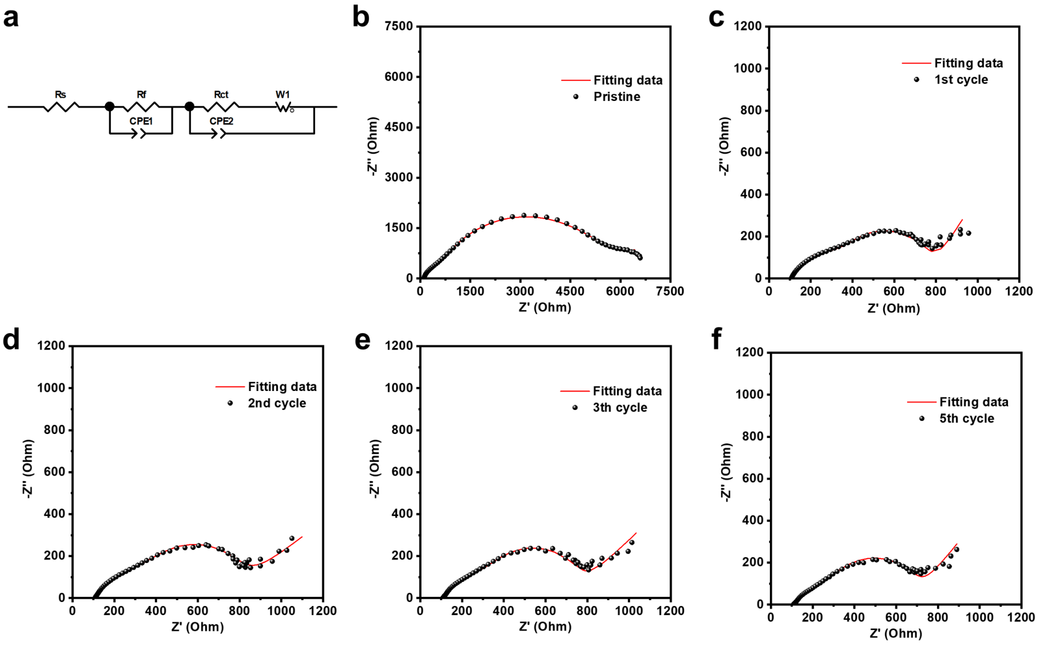

Supplementary Figure 19. (a) Equivalent circuit model; (b-f) EIS tests of K||Cu cells with the KDP electrolyte at -40°C and the Fitted Nyquist plots at different cycles. The corresponding impedance data is shown in **Supplementary Table 5**.

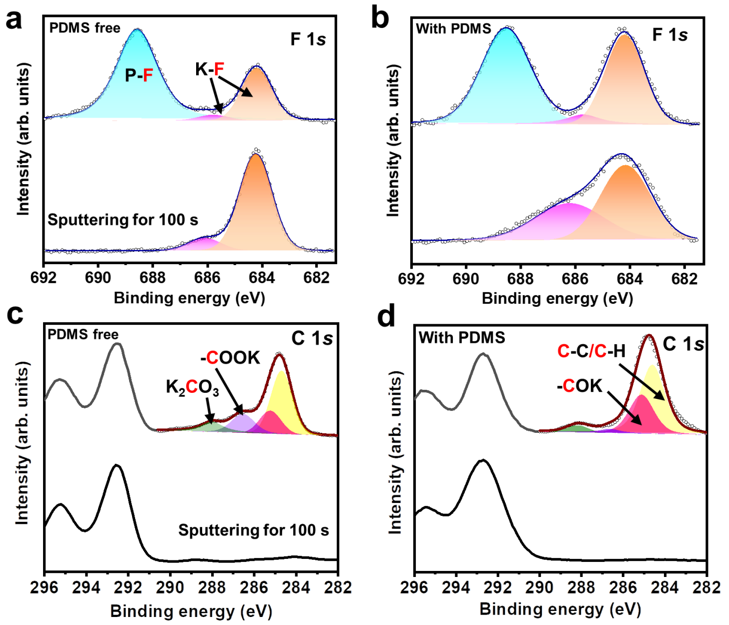

Supplementary Figure 20. In-depth XPS spectra of the cycled K anode at 25°C. F 1*s* in (a) KDP and (b) 0.4 M KPF_6-DME; C 1*s* in (c) KDP and (d) 0.4 M KPF_6-DME.

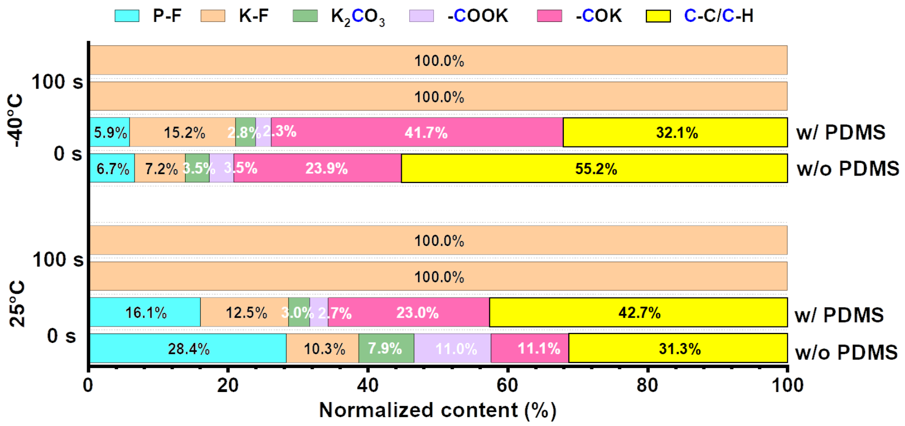

Supplementary Figure 21. Normalized ratios of different species in the SEI formed in different electrolytes and temperatures.

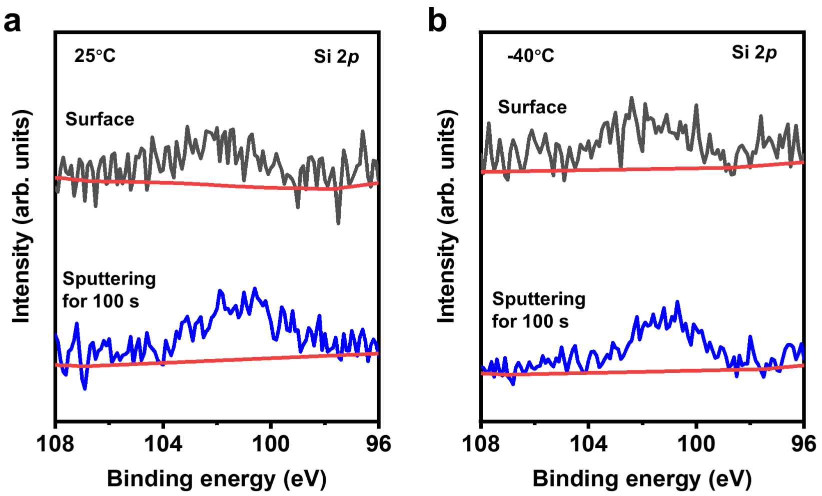

Supplementary Figure 22. In-depth Si 2*p* XPS spectra of the K anode cycled in the KDP electrolyte at (a) 25 and (b) -40°C, respectively.

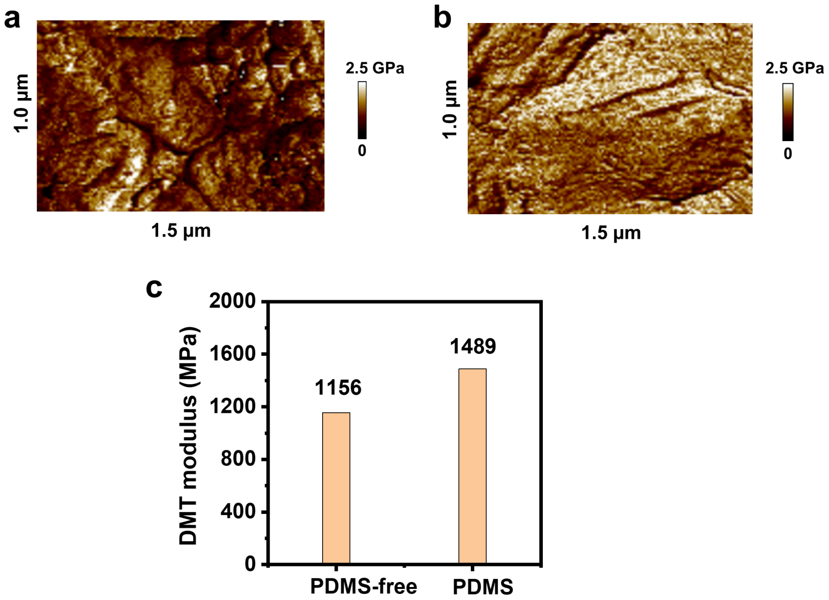

Supplementary Figure 23. DMT modulus mappings of SEI formed on K anodes with (a) the KDP electrolyte and (b) 0.4 M KPF_6-DME; (c) The corresponding average DMT modulus.

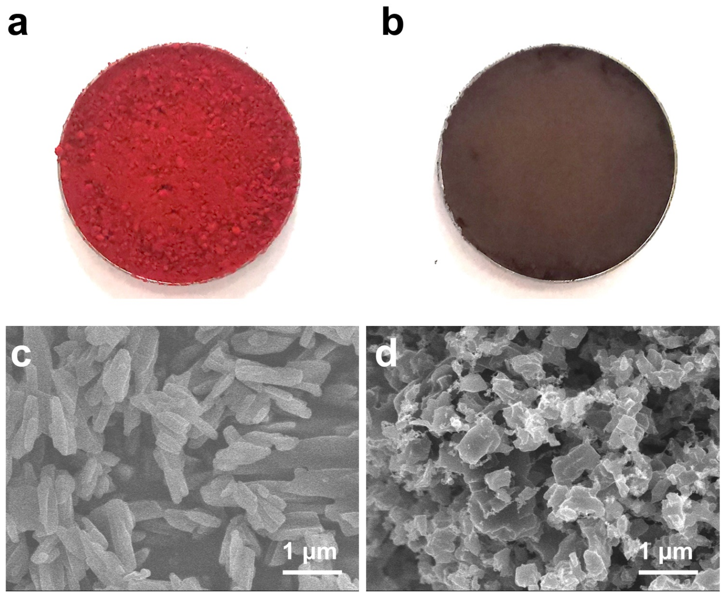

Supplementary Figure 24. The photos of (a) pristine PTCDA and (b) annealed PTCDA at 450°C for 4 h; (c, d) The corresponding SEM images.

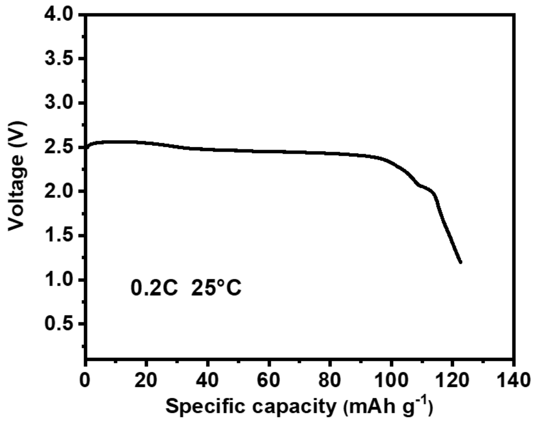

Supplementary Figure 25. The PTCDA cathode were prepotassiated in the K||PTCDA coin cell at 0.2C and 25°C.

The K||PTCDA exhibits a specific capacity of 125 mAh g^{-1}, approaching the theoretical capacity of 131 mAh g^{-1} for the PTCDA cathode. Then, the prepotassiated PTCDA was used to assemble anode-free Cu||KPTCDA full cells for further electrochemical tests.

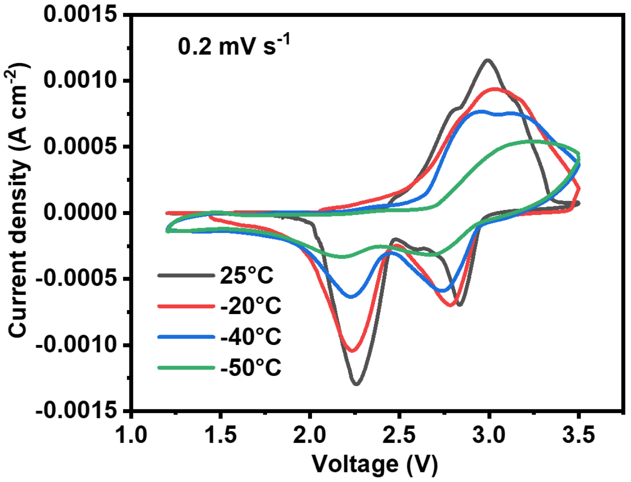

Supplementary Figure 26. CV curves of Cu||KPTCDA full coin cells over a temperature range from 25 to -40°C.

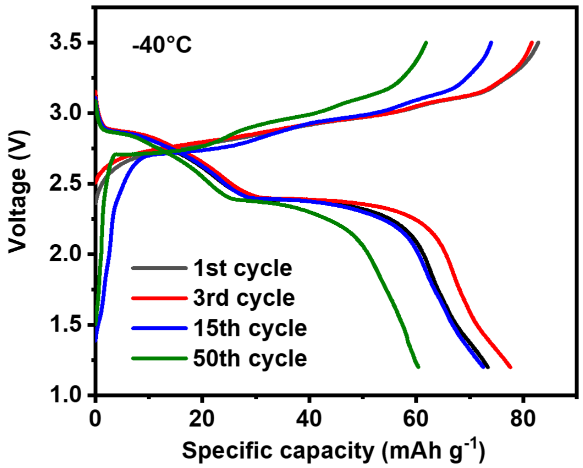

Supplementary Figure 27. Voltage profiles of Cu||KPTCDA full coin cells at different cycles.

The specific capacity at 3rd cycle is 77.5 mAh g^{-1}. Then, the energy density based on the total loading mass of the negative and positive electrodes can be calculated as 152 Wh kg^{-1}. Please refer to **Supplementary Table 6** for details.

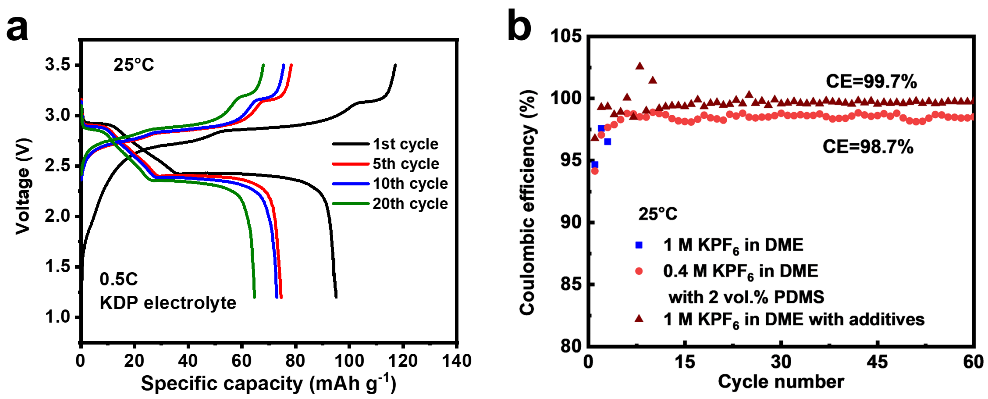

Supplementary Figure 28. (a) Voltage-specific capacity profiles of Cu||KPTCDA full cells at different cycles; (b) The K plating/stripping CE of K||Cu half cells with 3 electrolytes at 1.0 mA cm^{-2}/1 mAh cm^{-2}.

The Cu||KPTCDA batteries exhibit an initial discharge capacity of 95.1 mAh g^{-1} at 25°C with the KDP electrolyte, and the calculated energy density is 188 Wh kg^{-1} based on the total mass of the negative and positive electrodes (**Supplementary Table 6**). Besides, a capacity retention of 63% is observed after 20 cycles, which can be attributed to the imperfect reversibility of K plating/stripping at the anode side (average CE=98.7%). However, by introducing new additives, the CE of K||Cu half cells can be further improved to 99.7%, and it will be reported in the future work.

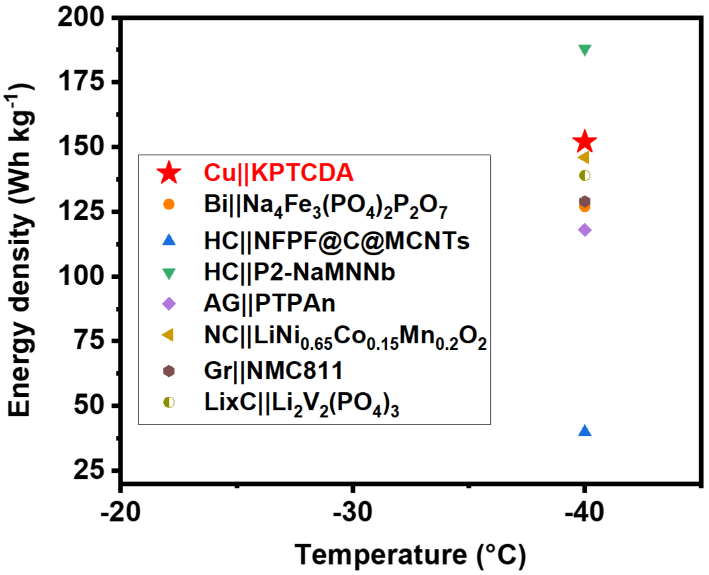

Supplementary Figure 29. Comparison of energy densities with Li/Na-ion full cells at -40°C. The energy densities were calculated based on the total loading mass of cathode and anode. Note: the data listed in **Supplementary Table 7** is used for the plot.

Supplementary Table 1. Comparison of the properties of typical alkali metal elements.

	Li	Na	K
Crust abundance (wt.%)	0.0017	2.3	1.5
Charge, *Z*	+1	+1	+1
Ionic radius, R (Å)	0.76	1.02	1.38
Cost of industrial grade metal (US$ ton^{-1})	100,000	3000	13,000
First ionization energy (KJ mol^{-1})	520.2	495.8	418.8
Charge density, *Z*/R	1.32	0.98	0.72

Note: Lewis acidity is assessed by the charge density.

Supplementary Table 2. The calculated coordination number in 0.4 M and 1 M KPF_6-DME at 233K.

	Coordination number at 233K	
	0.4 M KPF_6-DME	1 M KPF_6-DME
K-O (at 4.1 Å)	6.787	6.048
K-P (at 5.1 Å)	0.598	0.938
K-F (at 3.8 Å)	1.365	2.129

Supplementary Table 3. The corresponding impedance data obtained from K||Cu cells with the 0.4 and 1 M electrolyte at -40°C, respectively.

	**0.4 M**	**1 M**
Rs (Ω)	71	96
Fitting error	0.38%	0.41%
Rf (Ω)	96	128
Fitting error	5.1%	10.3%
CPE1-T ($\Omega^{-1}\cdot s^{n}$)	2.17E-5	1.31E-5
Fitting error	12.1%	5.68%
CPE1-P (unitless)	0.93	0.73
Fitting error	1.7%	1.89%
Rct (Ω)	759	1638
Fitting error	8.6%	10.89%
W1-R (Ω)	3963	2391
Fitting error	22.5%	155%
W1-T (s)	1010	56.3
Fitting error	90%	15.1%
W1-P (unitless)	0.21	0.51
Fitting error	7.2%	6.3%
CPE2-T ($\Omega^{-1}\cdot s^{n}$)	5.03E-5	1.54E-5
Fitting error	3.5%	4.5%
CPE2-P (unitless)	0.79	0.61
Fitting error	2.3%	3.3%

Supplementary Table 4. Performance comparison with other low-temperature K metal batteries.

	Areal capacity (mAh cm^{-2})	Rate (mA cm^{-2})	Overpotential (V)	Average CE (%)	CPC (mAh cm^{-2})	Temperature (℃)
Ref. 4	0.17	1	8.3	#	0.34	-40
Ref. 5	0.1	0.1	2	#	3	-30
Ref. 6	0.2	0.2	0.6	#	60	-20
**This work**	**1**	**4**	**0.13**	**99.80**	**500**	**-40**

Supplementary Table 5. The corresponding impedance data obtained from K||Cu cells with the KDP electrolyte at -40°C.

	**Pristine**	**After 1 cycle**	**After 2 cycles**	**After 3 cycles**	**After 5 cycles**
Rs (Ω)	108.1	107.1	107.8	108.2	107.7
Fitting error	0.54%	0.14%	1.43%	0.56%	1.13%
Rf (Ω)	397.2	225.7	271.9	235.1	229.1
Fitting error	14.7%	15.6%	11.2%	12.1%	8.9%
CPE1-T ($\Omega^{-1}\cdot s^{n}$)	6.49E-6	2.81E-5	1.76 E-4	1.41 E-4	2.41E-4
Fitting error	11.2%	25.2%	27.1%	15.6%	20.1%
CPE1-P (unitless)	0.86	0.75	1.036	0.63	0.62
Fitting error	2.7%	5.5%	6.1%	4.1%	3.8%
Rct (Ω)	4872.5	337.4	352.6	335.3	322.4
Fitting error	5.7%	7.6%	5.8%	3.4%	3.8%
W1-R (Ω)	1692	8000	3583	2233	2864
Fitting error	6.7%	50.7%	37.3%	28.1%	31.8%
W1-T (s)	2.9	218.2	623.3	210.3	213.5
Fitting error	17.3%	77.2%	87.2%	36.7%	53.1%
W1-P (unitless)	0.087	0.65	0.42	0.51	0.61
Fitting error	7.3%	0.43%	0.48%	0.71%	0.44%
CPE2-T ($\Omega^{-1}\cdot s^{n}$)	2.01E-5	6.16E-5	7.58E-5	1.512E-4	2.79E-4
Fitting error	2.8%	2.1%	3.5%	1.5%	1.0%
CPE2-P (unitless)	0.79	1.03	0.71	1.11	1.08
Fitting error	1.3%	0.9%	1.6%	1.0%	0.7%

Supplementary Table 6. The calculation of full-cell energy density.

**Parameters**	**Cell 1 (at -40℃)**	**Cell 2 (at -40℃)**
PTCDA:Super P:CMC	8:1:1	8:1:1
Total loading mass of cathode before prepotassiation/mg	9	6
Total loading mass of cathode after prepotassiation/mg	10.31	6.88
Total loading mass of anode/mg	0	0
Operating temperature/℃	-40	25
Operating voltage/V	2.39	2.41
Discharge capacity of Cu||KPTCDA cells/mAh	0.655	0.538
Specific capacity of Cu||KPTCDA cells/mAh g^{-1}	77.5	95.1
Energy density based on the total loading mass of cathode and anode/Wh kg^{-1}	152.6	188.4

Supplementary Table 7. Comparisons of the electrochemical performance between this work and the reported low-temperature rechargeable batteries.

Cell Configuration	Operating Temperature (°C)	Current rate (A g^{-1})	Average discharge voltage(V)	Specific energy (Wh kg^{-1})	Ref.
**Cu‖KPTCDA**	**-40**	**0.026**	**2.39**	**152**	**This work**
Bi‖ $Na_4Fe_3(PO_4)_2P_2O_7$	-40	0.02	2.3	127.5	Ref. 7
HC‖ NFPF@C@MCNTs	-40	0.037	2.1	39.4	Ref. 8
HC‖P2-NaMNNb	-40	0.037	3.2	188	Ref. 9
AG‖PTPAn	-40	0.01	2.5	118	Ref. 10
NC‖$LiNi_{0.65}Co_{0.15}Mn_{0.2}O_2$	-40	0.012	2.44	146.8	Ref. 11
Gr‖NMC811	-40	0.02	3.4	129	Ref. 12
LixC‖$Li_2V_2(PO_4)_3$	-40	0.017	3.92	139	Ref. 13

Note: the energy density is calculated based on the total mass of the negative and positive electrodes.

Supplementary References

1. Liu, J., Jiang, R., Wang, X., Huang, T. & Yu, A. The defect chemistry of $LiFePO_4$ prepared by hydrothermal method at different pH values. *J. Power Sources* **194**, 536-540 (2009).
2. Gao, F. & Tang, Z. Kinetic behavior of $LiFePO_4$/C cathode material for lithium-ion batteries. *Electrochimica Acta* **53**, 5071–5075 (2008).
3. Chen, X. et al. Phase transfer-mediated degradation of ether-based localized high-concentration electrolytes in alkali metal batteries. *Angew. Chem. Int. Ed.* **61**, e202207018 (2022).
4. Liu, S. et al. Manipulating the solvation structure of nonflammable electrolyte and interface to enable unprecedented stability of graphite anodes beyond 2 years for safe potassium-ion batteries. *Adv. Mater.* **33**, e2006313 (2021).
5. Wang, J., Yan, W. & Zhang, J. High area capacity and dendrite-free anode constructed by highly potassiophilic Pd/Cu current collector for low-temperature potassium metal battery. *Nano Energy* **96**, 107131 (2022).
6. Yang, Q., Ding, Y. & He, G. An amalgam route to stabilize potassium metal anodes over a wide temperature range. *Chem. Commun.* **56**, 3512-3515 (2020).
7. Li, Z. et al. Sodium-ion battery with a wide operation-temperature range from −70 to 100 °C. *Angew. Chem. Int. Ed.* **61**, e202116930 (2022).
8. Cao, Y. et al. Pilot-scale synthesis sodium iron fluorophosphate cathode with high tap density for a sodium pouch cell. *Small* **18**, (2022).
9. Shi, Q. et al. Niobium-doped layered cathode material for high-power and low-temperature sodium-ion batteries. *Nat. Commun.* **13**, 3205 (2022).
10. Chen, J. et al. A desolvation-free sodium dual-ion chemistry for high power density and extremely low temperature. *Angew. Chem. Int. Ed.* **60**, 23858-23862 (2021).
11. Yang, Y. et al. Rechargeable $LiNi_{0.65}Co_{0.15}Mn_{0.2}O_2$||graphite batteries operating at −60 °C. *Angew. Chem. Int. Ed.* **61**, e202209619 (2022).
12. Nan, B. et al. Enhancing Li^+ transport in NMC811||graphite lithium-ion batteries at low temperatures by using low-polarity-solvent electrolytes. *Angew. Chem. Int. Ed.* **61**, e202205967 (2022).
13. Liu, Y., Yang, B., Dong, X., Wang, Y. & Xia, Y. A simple prelithiation strategy to build a high-rate and long-life lithium-ion battery with improved low-temperature performance. *Angew. Chem. Int. Ed.* **56**, 16606-16610 (2017).
